# The adverse effects of long-term exposure to anticholinergics among people with intellectual disabilities: a scoping review

**DOI:** 10.12688/hrbopenres.13599.1

**Published:** 2022-09-30

**Authors:** Lamya Al Shuhaimi, Martin Henman, Philip McCallion, Mary McCarron, Maire O'Dwyer

**Affiliations:** 1 School of Pharmacy and Pharmaceutical Sciences and IDS-TILDA School of Nursing and Midwifery, Trinity College Dublin, Dublin, D02 PN40, Ireland; 2 School of Social Work, Temple University, Philadelphia, PA, 19122, USA; 3 Trinity Centre for Ageing and Intellectual Disability, Trinity College Dublin, Dublin, D01 W596, Ireland

**Keywords:** anticholinergic burden, intellectual disability, long-term adverse effects, older adults

## Abstract

**Background:** Older adults with intellectual disability are exposed to a higher anticholinergic burden compared to general older adults. This is due to a higher rate of both mental and neurological disorders among people with intellectual disability. The use of medications with a high anticholinergic burden is associated with adverse effects including daytime dozing, constipation and higher dependence level in the Barthel index for measuring activities of daily living. This scoping review aims to map and examine the existing research on physical and cognitive adverse effects associated with the long-term impact of anticholinergics among people with intellectual disabilities.

**Methods: **The search was conducted in: PubMed, Cochrane library, EMBASE, Medline, Science Direct, CINAHL Complete and PsycINFO. Preliminary studies, grey literature, and conference papers were searched in related electronic databases. The search terms included terms related to ‘anticholinergic’, ‘long-term exposure’, ‘intellectual disability’ and ‘adverse drug reaction’ with Boolean operator ‘and’. Studies with at least three months’ exposure to anticholinergics were included. The search was restricted to research papers on people with intellectual disability aged 40 or over and publication in the English language only. Initially, it was conducted in May and June 2021 and covered the publication period between 1970 and 2021. It was re-run in October 2021.

**Results: **The conducted search provided 509 records of publications and grey literature. Duplicates were removed using EndNote 20 and resulted in 432 remaining records. Then, 426 further records were excluded because they were deemed irrelevant, or non-longitudinal studies or conducted on different populations. Only six full articles were retrieved to assess their eligibility and all were excluded due to different study populations. This resulted in no studies meeting the stated inclusion criteria.

**Conclusions: **Further research is urgently required to examine the long-term adverse effects associated with higher anticholinergic scores among older people with intellectual disability.

## Introduction

Medicines with anticholinergic (AC) activity are widely used among older adults due to their potential clinical benefits in managing a wide range of medical conditions ^1^. These medicines are usually used for peptic ulcer disorders, irritable bowel syndrome, urinary disorders, Parkinson’s disease, neurological diseases, psychiatric conditions and as anaesthetic agents.

There are well-recognized adverse effects associated with the use of AC which include: dry, pale and cool skin, dry mouth with difficulty beginning to speak, urinary diseases, blurred vision, unsteady movement and falls, anxiety, tachycardia, cardiac arrhythmias, etc ^2^. In addition, there are several central anticholinergic adverse effects associated with anticholinergic drugs that can cross the blood brain barrier such as memory impairment, agitation, hallucination, delirium, confusion and disorientation. 

In certain medical conditions that are usually associated with aging, the benefits of AC use are greater than their risks; therefore, it is inevitable and appropriate that they be used ^1^. These conditions include: psychiatric disorders (such as bipolar disorder, obsessive-compulsive disorders, severe insomnia, severe anxiety diseases, drug-induced acute dystonia, secondary Parkinsonism) and non-psychiatric conditions (such as gastroesophageal reflux disease, irritable bowel syndrome, urinary incontinence, neuropathic pain, cardiovascular disorders, muscle spasms and low back pain).

People with intellectual disabilities experience a higher incidence of morbidities, by 2.5 times, compared to the general population ^3^. They have a higher incidence of some diseases such as dementia, dental disorders, psychiatric diseases, osteoporosis and epilepsy. Furthermore, prevalence of polypharmacy is higher in people with an intellectual disability, and they are described as one of the most medicated groups of the population. A study conducted in Ireland has identified that older adults with intellectual disability are exposed to excessive polypharmacy (10 medications or more) at rates 10 times higher than the general population ^3^. Moreover, they are exposed to a high anticholinergic burden ^4, 5^ due to the high prevalence of mental disorders and neurological disorders ^6, 7^. Furthermore, a Scottish cohort study reported that anticholinergic burden was relatively high among all age groups of people with intellectual disability including adults and older adults (17–94 years old) ^5^. Notably, the greatest anticholinergic burden was observed in people aged ≥ 55 years and in females.

High anticholinergic burden was associated with a higher likelihood of reporting constipation, daytime dozing and use of multiple laxatives in the elderly with intellectual disabilities ^4^. Another study reported that a higher Drug Burden Index (DBI) was associated with higher dependence scores in the Barthel index for measuring activities of daily living in older adults with intellectual disabilities ^8^.

The long-term impact of anticholinergics has been examined by some studies among the general geriatric population. These studies have reported an association between long-term cumulative high anticholinergic burden and poorer physical function ^9– 11^, cognitive impairment ^9– 13^, and a higher incident of dementia ^14^. Another study found that high anticholinergic burden can predict mortality among elderly patients discharged from hospital with a high Geriatric Depression Scale (GDS) score ^15^.

According to our preliminary research, there has been no or limited research exploring the long-term impact of anticholinergics among older adults with an intellectual disability. Therefore, this scoping review aims to map and examine the existing research literature on physical and cognitive adverse effects associated with the long-term impact of anticholinergics among people with intellectual disabilities.

## Methods

This scoping review followed the framework proposed by Arksey and O’Malley ^16^ and its developed version by Levac ^17^. Moreover, the Joanna Briggs Institute reviewer’s manual ^18^ and the Preferred Reporting Items for Systematic reviews and Meta-Analysis extension for Scoping Reviews (PRISMA-ScR) ^19^ were also followed. Accordingly, there were six stages in conducting the review:

1. Identification of the research question
2. Identification of relevant studies
3. Selection of studies
4. Data charting
5. Collating, summarising and reporting the results
6. Consultation

A protocol ^20^ of this scoping review was published previously and provides more details of the methods used. ( https://doi.org/10.12688/hrbopenres.13266.1)

### Stage 1: Identification of the research question

As per our best knowledge, there was no research exploring the adverse effects associated with the long-term exposure to anticholinergics among older adults with intellectual disabilities. This scoping review was therefore conducted to answer the following question:

What are the adverse effects on cognitive and physical outcomes of long-term exposure to medications with anticholinergic activity among older adults with intellectual disabilities?

As a long-term goal, this review aimed to produce findings likely to enhance prescribing patterns among this vulnerable group of people.

### Stage 2: Identification of relevant studies

The search was conducted in seven electronic databases: PubMed, Cochrane library, EMBASE, Medline, Science Direct, CINAHL Complete and PsycINFO. Additionally, preliminary studies, grey literature, and conference papers were searched in Google Scholar, The Turning Research Into Practice (TRIP) database, ClinicalTrials.gov, PROSPERO, EU Clinical Trial Register, Open Grey, the WHO International Clinical Trials Registry Platform search portal (ICTRP), International Standard Randomised Controlled Trial Number (ISRCTN) registry, Chinese Clinical Trial Registry (ChiCTR), Australian New Zealand Clinical Trials Registry (ANZCTR), Pan African Clinical Trials Registry (PACTR) and Clinical Trials Registry—India (CTRI).

The search terms used to conduct the electronic search consisted of four terms/phrases and these were ‘anticholinergic’, ‘long-term exposure’, ‘intellectual disability’ and ‘adverse drug reaction’. The keywords and Medical Subject Headings (MeSH) terms for each phrase was searched with Boolean operator ‘AND’. Table 1 provides the keywords used to conduct the search.

**Table 1.** Search keywords: the table illustrates the terms used for conduction of the search using the electronic databases.

Anticholinergic	Long-term Exposure	Intellectual Disability	Adverse Drug Reaction
‘anticholinergic burden’ OR ‘anticholinergic exposure’ OR ‘anticholinergic’ OR ‘cholinergic antagonist’ OR ‘antimuscarinic drugs’ OR ‘ muscarinic antagonist’ OR ‘ anticholinergic’ OR ‘ anticholinergic agent’ OR ‘ antimuscarinic’ OR ‘ antimuscarinic agent’ OR ‘cholinergic blocking agent’ OR ‘acetylcholine antagonist’ OR ‘cholinergic receptor antagonist’ OR ‘cholinergic antagonists’ OR ‘cholinolytics’	‘chronic use’ OR ‘long-term use’ OR ‘ ≥ 3 months’	‘cognitive impairment’ OR ‘intellectual disabilities’ OR ‘learning disabilities’ OR ‘developmental disabilities’ OR ‘mentally disabled persons’ OR ‘handicap’ OR ‘Down Syndrome’ OR ‘mental retardation’ OR ‘intellectual development disorders’ OR ‘psychosocial mental retardation’ OR ‘mental deficiency’ OR ‘mentally disabled’ OR ‘mentally handicapped’ OR ‘persons with intellectual disability’ OR ‘mentally retarded’	‘adverse effect’ OR ‘drug toxicity’ OR ‘adverse outcomes’ OR ‘side effects’ OR ‘drug related side effects’ and ‘adverse reactions’ OR ‘side effects of drugs’ OR ‘drug side effects’ OR ‘adverse drug reactions’ OR ‘adverse drug event’ OR ‘anticholinergic toxicity’ OR ‘anticholinergic effect’ OR ‘anticholinergic syndrome’ OR ‘peripheral anticholinergic syndrome’ OR ‘central anticholinergic syndrome’ OR ‘anticholinergic toxicity’ OR ‘anticholinergic effect’ OR ‘cognitive function’ OR ‘cognitive disorder’ OR ‘cognitive impairment’ OR ‘ dementia’ OR ‘delirium’ OR ‘physical function’ OR ‘ physical activity’ OR ‘frailty’ OR ‘falls’ OR ‘accidental falls’ OR ‘hip fracture’ OR ‘mortality’ OR ‘death’ OR ‘constipation’ OR ‘urinary incontinence’

There is no identified age range to describe older adults with intellectual disability. However, most of the published papers considered that older adults with intellectual disability are those aged ≥ 40 years old ^4, 7, 8, 21^. Therefore, the search was restricted to research papers on people with intellectual disability aged 40 or over and publication in English language only. It was conducted in May and June 2021 (2021-05-29, 2021-06-10, 2021-06-14) and covered the publication period between 1970 and 2021. The search was re-run again in October 2021.

### Stage 3: Selection of studies

Studies were included according to the inclusion and exclusion criteria. The titles and abstracts were screened by team member ‘LAA’ to assess their eligibility according to the stated inclusion criteria. A second reviewer (MO’D) screened the retrieved articles to independently confirm their eligibility. A Microsoft Word (Version 16.63.1) document was prepared that included the details of search records such as keywords, MeSH terms, search limitations, results and number of included articles for each electronic database used, as summarized above.

**Figure 1.**
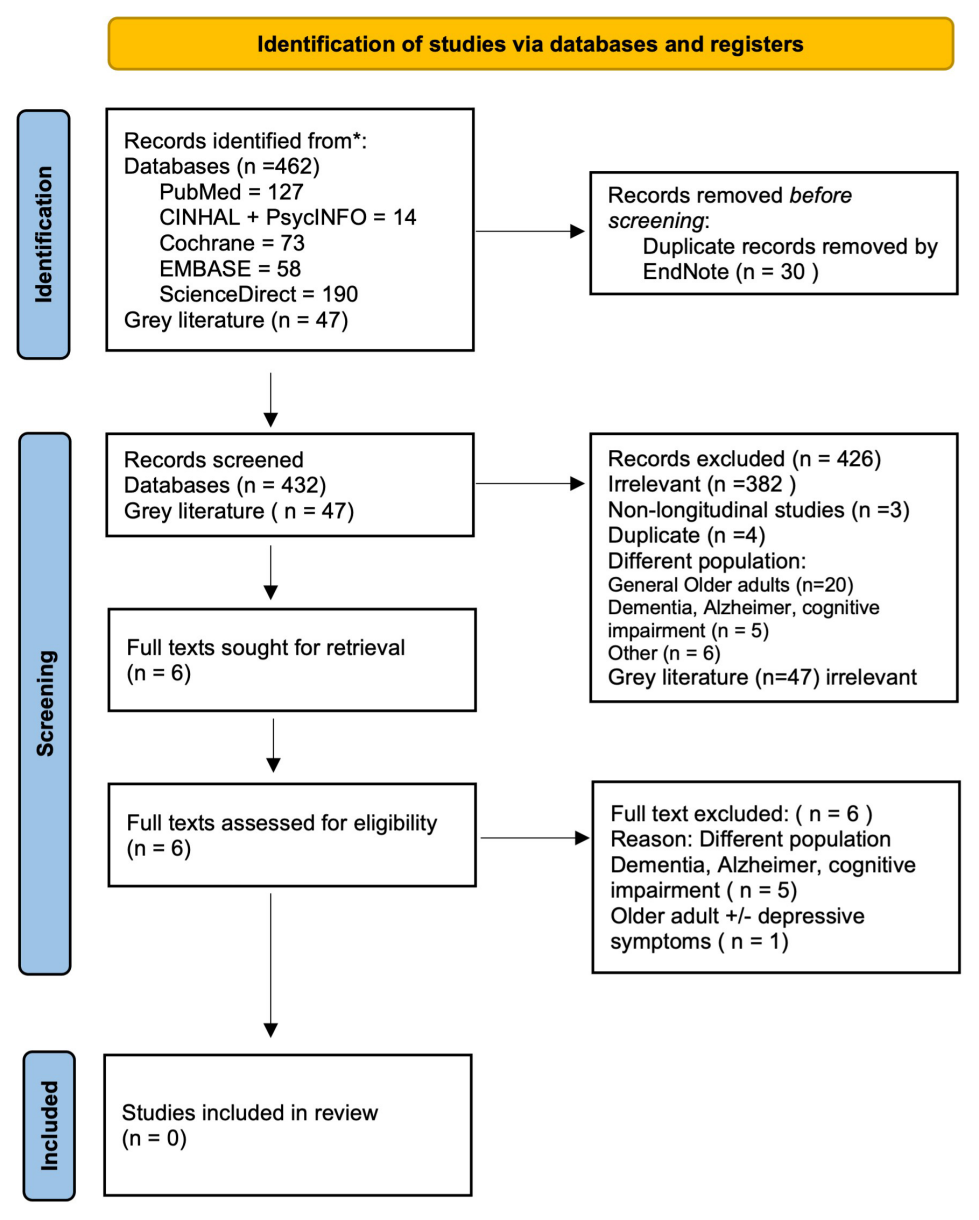
PRISMA flow chart illustrating the process of study selection.

***Inclusion criteria***

Study design

All designs and types of study were included.

Study population

Only studies on people with intellectual disability were included. Intellectual disability usually occurs prior to age 18 and it is defined as “a disability characterized by significant limitations in both intellectual functioning and adaptive behaviour, which covers many everyday social and practical skills.” [ https://www.aaidd.org/]. Various types and stages of intellectual disability are included in this scoping review.

Furthermore, the review only included studies on older adults aged 40 or more with intellectual disability. Children and adults with intellectual disability who were aged less than 40 years were excluded from the review.

Intervention

‘Long-term use’ or ‘Chronic use’ of medicines has been defined as being on a medicine for at least three months or longer [ https://www.medicinenet.com/]. Therefore, the review included all studies that examined the adverse outcomes associated with the use of anticholinergics for a period of three months or longer. There was no restriction on tools used to identify anticholinergic burden.

Context

The review included research papers in all different care settings where people with intellectual disability can live, including institutions, nursing homes, community group homes, with families or independently. 

Outcomes

This scoping review examined both cognitive and physical outcomes associated with the long-term exposure to anticholinergics in older adults with intellectual disability. The physical and cognitive outcomes include all of the reported and measured central and peripheral adverse effects associated the long-term use of these medications. There are no restrictions of tools used to report or measure the adverse outcomes.

figure 1 illustrates the process of study selection according to the Preferred Reporting Items for Systematic Reviews and Meta-Analyses extension for Scoping Reviews (PRISMA-ScR).

### Stage 6: Consultation

Expert members of The Royal College of Psychiatrists of Ireland (Intellectual disability subgroup), Providers in Ireland: Daughters of Charity Service, Down Syndrome Ireland, National Intellectual Disability Memory Service, Tallaght Hospital and the International Association for the Scientific Study of Intellectual and Developmental Disabilities (IASSIDD) were consulted verbally or by email on the scoping review research question and its findings.

## Differences between protocol and review

### Stage 2: Identification of relevant studies

Open Grey was also searched for unpublished articles. It wasn’t included in the previously published review protocol.

### Stages 4 and 5: Data charting, collating, summarising and reporting the results

A data extraction form was developed to chart information from the included studies. The form was designed to include key information from each included article such as title, aim, population, results, conclusion and implications. However, none of the studies fitted the stated inclusion criteria. Therefore, there was no data to be charted, collated, summarized and reported.

## Results

### Description of studies

The initial search conducted in May and June 2021 was re-run on 2021-10-05 and 2021-10-08 and provided 509 records of both publications and grey literature. Duplicates were removed by using EndNote 20 (RRID:SCR_014001) and resulted in 432 remaining records. Then, the records were screened for relevance based on the titles and abstracts by two members of the search team. After that, 426 further records were excluded because they were deemed irrelevant, or non-longitudinal studies or conducted on different populations. Only six full articles were retrieved to assess their eligibility to be included in the stated inclusion criteria. Two members (MO’D, LAA) of the research team assessed the remaining six records for their relevance and all were excluded due to different studied populations. This resulted in no studies meeting the stated inclusion criteria.

### Consultation findings

Representative members from The Royal College of Psychiatrists of Ireland (Intellectual disability subgroup), Down Syndrome Ireland and the International Association for the Scientific Study of Intellectual and Developmental Disabilities (IASSIDD) replied to our consultation request. The representatives highlighted the reasons such prescribing occurs, the particular risk for persons with Down syndrome, the important role played by the longitudinal study findings and the acceptance that research in this area must become a scientific priority. No concerns were noted about the review findings.

## Discussion

As far as we are aware, this is the first scoping review to examine the adverse effects of long-term exposure to anticholinergics among older adults with intellectual disability. This scoping review aimed to map and examine the available research. However, no studies were identified that met the inclusion criteria of the review and an empty review (a review with no studies eligible for inclusion based on the inclusion criteria ^22^.) has been reported. This fits a pattern of 15 empty reviews out of 81 reported systematic reviews conducted on people with developmental, psychosocial and learning problems ^23^.

Empty reviews help to identify important gaps in knowledge and to stimulate researchers towards topic areas that need to be studied ^24^. According to studies that were screened in this scoping review, there is some limited identification of the prevalence of anticholinergic burden and the associated short-term adverse effects among people with intellectual disability. In addition, the long-term adverse impact of anticholinergics has been examined among the general population ^9– 15^, patients with cognitive impairments, dementia and Alzheimer’s disease ^25– 29^. That there are few or no studies conducted among people with intellectual disability in an area where there are published studies for the general population suggests people with intellectual disabilities still remain mostly excluded from participating in clinical research ^6^. It is of further concern that there is therefore a restricted evidence base for safe use of these medicines among this vulnerable group of the population.

## Conclusions

In conclusion, further research is urgently required to examine the long-term adverse effects associated with higher anticholinergic scores among older people with intellectual disability.

## Data Availability

Open Science Framework (OSF): Underlying data for ‘The adverse effects of long-term exposure to anticholinergics among people with intellectual disabilities: a scoping review’. https://doi.org/10.17605/OSF.IO/CVZX3 ^19^. Open Science Framework (OSF): PRISMA-ScR checklist for ‘The adverse effects of long-term exposure to anticholinergics among people with intellectual disabilities: a scoping review’. https://doi.org/10.17605/OSF.IO/CVZX3 ^19^. Data are available under the terms of the Creative Commons Zero “No rights reserved” data waiver (CC0 1.0 Public domain dedication).

## References

[1] López-Álvarez J Sevilla-Llewellyn-Jones J Agüera-Ortiz L : Anticholinergic Drugs in Geriatric Psychopharmacology. *Front Neurosci.* 2019;13:1309. 10.3389/fnins.2019.01309 31866817 PMC6908498

[2] Mintzer J Burns A : Anticholinergic side-effects of drugs in elderly people. *J R Soc Med.* 2000;93(9):457–462. 10.1177/014107680009300903 11089480 PMC1298101

[3] O'Dwyer M Peklar J McCallion P : Factors associated with polypharmacy and excessive polypharmacy in older people with intellectual disability differ from the general population: a cross-sectional observational nationwide study. *BMJ Open.* 2016;6(4):e010505. 10.1136/bmjopen-2015-010505 27044582 PMC4823458

[4] O'Dwyer M Maidment I Bennett K : Association of anticholinergic burden with adverse effects in older people with intellectual disabilities: an observational cross-sectional study. *Br J Psychiatry.* 2016;209(6):504–510. 10.1192/bjp.bp.115.173971 27660331

[5] Ward LM Stanley B Greenlaw N : Risk of anticholinergic burden in adults with intellectual disabilities: a Scottish retrospective cohort study of *n* = 17 220. *J Intellect Disabil Res.* 2021;65(9):813–830. 10.1111/jir.12861 34169610

[6] O’Dwyer M McCallion P McCarron M : Measuring drug burden in older adults with intellectual disabilities: Critical issues for consideration in finding the optimal measure to improve safety of medicines use. *Expert Opin Drug Saf.* 2020;19(6):649–652. 10.1080/14740338.2020.1751119 32241202

[7] De Vreese L Mantesso U De Bastiani E : Anticholinergic burden in adult and elderly people with intellectual disabilities: Results from an Italian multicenter cross-sectional study. *PLoS One.* 2018;13(10):e0205897. 10.1371/journal.pone.0205897 30379948 PMC6209221

[8] O'Connell J Burke É Mulryan N : Drug burden index to define the burden of medicines in older adults with intellectual disabilities: An observational cross-sectional study. *Br J Clin Pharmacol.* 2018;84(3):553–567. 10.1111/bcp.13479 29193284 PMC5809360

[9] Wouters H Hilmer SN Gnjidic D : Long-Term Exposure to Anticholinergic and Sedative Medications and Cognitive and Physical Function in Later Life. *J Gerontol A Biol Sci Med Sci.* 2019;75(2):357–365. 10.1093/gerona/glz019 30668633

[10] Han L Agostini JV Allore HG : Cumulative Anticholinergic Exposure Is Associated with Poor Memory and Executive Function in Older Men. *J Am Geriatr Soc.* 2008;56(12):2203–2210. 10.1111/j.1532-5415.2008.02009.x 19093918 PMC3952110

[11] Brombo G Bianchi L Maietti E : Association of Anticholinergic Drug Burden with Cognitive and Functional Decline Over Time in Older Inpatients: Results from the CRIME Project. *Drugs Aging.* 2018;35(10):917–924. 10.1007/s40266-018-0584-9 30191516

[12] Cai X Campbell N Khan B : Long-term anticholinergic use and the aging brain. *Alzheimers Dement.* 2012;9(4):377–385. 10.1016/j.jalz.2012.02.005 23183138 PMC3674201

[13] Jamsen KM Gnjidic D Hilmer SN : Drug Burden Index and change in cognition over time in community-dwelling older men: the CHAMP study. *Ann Med.* 2016;49(2):157–164. 10.1080/07853890.2016.1252053 27763767

[14] Gray SL Anderson ML Dublin S : Cumulative Use of Strong Anticholinergics and Incident Dementia. *JAMA Intern Med.* 2015;175(3):401–7. 10.1001/jamainternmed.2014.7663 25621434 PMC4358759

[15] Corsonello A Cozza A D'Alia S : The excess mortality risk associated with anticholinergic burden among older patients discharged from acute care hospital with depressive symptoms. *Eur J Intern Med.* 2019;61:69–74. 10.1016/j.ejim.2018.11.004 30449478

[16] Arksey H O'Malley L : Scoping studies: towards a methodological framework. *Int J Soc Res Methodol.* 2005;8(1):19–32. 10.1080/1364557032000119616

[17] Levac D Colquhoun H O'Brien KK : Scoping studies: advancing the methodology. *Implement Sci.* 2010;5(1):69. 10.1186/1748-5908-5-69 20854677 PMC2954944

[18] The Joanna Briggs Institute: The Joanna Briggs Institute Reviewers’ manual 2015 - methodology for JBI scoping reviews.Adelaide, South Australia,2015. Reference Source

[19] Shuahimi L : The adverse effects of long-term exposure to anticholinergics among people with intellectual disabilities: a scoping review. Open Science Framework.[Dataset].2022. 10.17605/OSF.IO/CVZX3 PMC10308137 37396688

[20] Al Shuhaimi L Henman M McCallion P : The impact of long-term exposure to anticholinergics among people with intellectual disabilities: a scoping review protocol [version 1; peer review: 1 approved with reservations]. *HRB Open Res.* 2021;4:62. 10.12688/hrbopenres.13266.1 PMC10308137 37396688

[21] O’Connell J Henman MC Burke É : Association of Drug Burden Index with grip strength, timed up and go and Barthel index activities of daily living in older adults with intellectual disabilities: an observational cross-sectional study. *BMC Geriatr.* 2019;19(1):173. 10.1186/s12877-019-1190-3 31234775 PMC6591943

[22] Lang A Edwards N Fleiszer A : Empty systematic reviews: hidden perils and lessons learned. *J Clin Epidemiol.* 2007;60(6):595–597. 10.1016/j.jclinepi.2007.01.005 17493517

[23] Yaffe J Montgomery P Hopewell S : Empty Reviews: A Description and Consideration of Cochrane Systematic Reviews with No Included Studies. *PLoS One.* 2012;7(5):e36626. 10.1371/journal.pone.0036626 22574201 PMC3344923

[24] Gray R : Empty systematic reviews: Identifying gaps in knowledge or a waste of time and effort? *Nurse Author Ed.* 2021;31(2):42–44. 10.1111/nae2.23

[25] Fox C Livingston G Maidment ID : The impact of anticholinergic burden in Alzheimer's Dementia-the Laser-AD study. *Age Ageing.* 2011;40(6):730–735. 10.1093/ageing/afr102 21926432

[26] Ah YM Suh Y Jun K : Effect of anticholinergic burden on treatment modification, delirium and mortality in newly diagnosed dementia patients starting a cholinesterase inhibitor: A population‐based study. *Basic Clin Pharmacol Toxicol.* 2019;124(6):741–748. 10.1111/bcpt.13184 30511428

[27] Naharci MI Cintosun U Ozturk A : Effect of anticholinergic burden on the development of dementia in older adults with subjective cognitive decline. *Psychiatry and Clinical Psychopharmacology.* 2017;27(3):263–270. 10.1080/24750573.2017.1358130

[28] Dyer AH Murphy C Segurado R : Is Ongoing Anticholinergic Burden Associated With Greater Cognitive Decline and Dementia Severity in Mild to Moderate Alzheimer's Disease? *J Gerontol A Biol Sci Med Sci.* 2020;75(5):987–994. 10.1093/gerona/glz244 31613323

[29] Green AR Reifler LM Bayliss EA : Drugs Contributing to Anticholinergic Burden and Risk of Fall or Fall-Related Injury among Older Adults with Mild Cognitive Impairment, Dementia and Multiple Chronic Conditions: A Retrospective Cohort Study. *Drugs Aging.* 2019;36(3):289–297. 10.1007/s40266-018-00630-z 30652263 PMC6386184

