# Supplementary material for: The adverse effects of long-term exposure to anticholinergics among people with intellectual disabilities: a scoping review
Source: HRB Open Res. 2022 Sep 30;5:63. [Version 1] doi: 10.12688/hrbopenres.13599.1 (PMC10308137; doi:10.12688/hrbopenres.13599.1)
Supplement: Peer review history

# Peer review: Reviewer response for version 1

**DOI:** 10.21956/hrbopenres.14858.r34306

**Published:** 2023-06-28

**Authors:** David Harley

**Affiliations:** 1 UQ Centre for Clinical Research, The University of Queensland, Herston, Qld, Australia

This is a well written review dealing with an important issue in developmental disability medicine. As the authors point out, while it is an empty review this, again, highlights the importance of research on prescribing, particularly polypharmacy and drugs with significant adverse effects, to people with intellectual and developmental disability.

 I have no substantive criticisms of the review. Below I note some minor areas for improvement of the manuscript.

- In the introduction, change "Parkinson’s disease, neurological diseases" to "neurological diseases including Parkinson's".
- In the sentence stating that people with intellectual disability experience "a higher incidence of morbidities, by 2.5 times, compared to the general population", I suspect "incidence" should be "prevalence". Please check and correct if appropriate.
- In the sentence "Another study found that high anticholinergic burden can predict mortality among elderly patients discharged from hospital with a high Geriatric Depression Scale (GDS) score "predict" should be replaced with a word or phrase implying association rather than causation.
- I don't think team member initials need to be in inverted commas (only occurs once for member LAA).

Are the rationale for, and objectives of, the Systematic Review clearly stated?

Yes

Is the statistical analysis and its interpretation appropriate?

Yes

Are sufficient details of the methods and analysis provided to allow replication by others?

Yes

Are the conclusions drawn adequately supported by the results presented in the review?

Yes

Reviewer Expertise:

Adult developmental disability medicine, focussing particularly on psychotropic prescribing.

I confirm that I have read this submission and believe that I have an appropriate level of expertise to confirm that it is of an acceptable scientific standard.

# Peer review: Reviewer response for version 1

**DOI:** 10.21956/hrbopenres.14858.r33305

**Published:** 2023-01-09

**Authors:** Carrie Stewart

**Affiliations:** 1 Ageing Clinical and Experimental Research (ACER) Team, Institute of Applied Health Sciences, University of Aberdeen, Aberdeen, UK

This is well written and thorough systematic review in a novel research area and I thank the authors for their efforts in publishing this empty review. The search strategy appears thorough, has included grey literature and I am satisfied that the conclusions (empty review) are justifiable and reported within correct reporting guidelines.

Are the rationale for, and objectives of, the Systematic Review clearly stated?

Yes

Is the statistical analysis and its interpretation appropriate?

Not applicable

Are sufficient details of the methods and analysis provided to allow replication by others?

Yes

Are the conclusions drawn adequately supported by the results presented in the review?

Yes

Reviewer Expertise:

Deprescribing; ageing health

I confirm that I have read this submission and believe that I have an appropriate level of expertise to confirm that it is of an acceptable scientific standard.
